# Freedom of Medical Opinion Illustrated

**Published:** 1884-05

**Authors:** 


FREEDOM OF MEDICAL OPINION ILLUSTRATED.—Dr. A. M. Cushing, as President of the Massachusetts State "Homœopathic" Medical Society, delivered the usual annual address. The address was *rejected* by the publication committee, the sole offense being, as the author states, because he *dared* to declare that homœopaths should practice Homœopathy!! This was *more* than the M. S. H. Society bargained for, hence its rejection, by which this publication committee paid Dr. Cushing the greatest honor in their power to pay any one.

